# Experimental study on the bearing capacity of PZ shape composite dowel shear connectors with elliptical holes

**DOI:** 10.1038/s41598-022-06387-1

**Published:** 2022-02-14

**Authors:** Pingming Huang, Junlong He, Fanlei Kong, Kuihua Mei, Xiaolong Li

**Affiliations:** School of Highway, Chang’an University, Xi’an, 710064 Shaanxi China

**Keywords:** Civil engineering, Mechanical engineering

## Abstract

The push-out test was carried out in order to study the shear performance of perfobond ribs (PBL) and puzzle (PZ) shape composite dowel shear connectors which are used in corrugated steel–concrete composite bridge decks. By considering the influencing factors of transversal reinforcement, elliptical holes and bonding friction forces, eight specimens were designed and prepared. The load–slip curves were obtained, and the static mechanical properties, including bearing capacity, slip, shear stiffness, and ductility factor, were analyzed. The formula of bearing capacity and load–slip curve were proposed. The results showed a clear deformation of the transversal reinforcement of both the two types of shear connectors. The dominating failure mode of both the shear connectors was the double plane shearing of the concrete dowel. The load–slip curves of the two types of shear connectors followed the same trend. The PZ shape composite dowel performed better in ultimate bearing capacity, ductility, and shear stiffness than the PBL shear connector. Elliptical holes in a PZ shape composite dowel significantly increased the ultimate bearing capacity. The shear stiffness increased with transversal reinforcement and reduced with elliptical holes. For PBL shear connectors, the strain change in the C1 area near the loading point was most apparent. While, the A1 and B1 areas were stress concentration locations for PZ shape composite dowel shear connectors. The two formulas were in good agreement with the test results.

## Introduction

**Figure 1:**
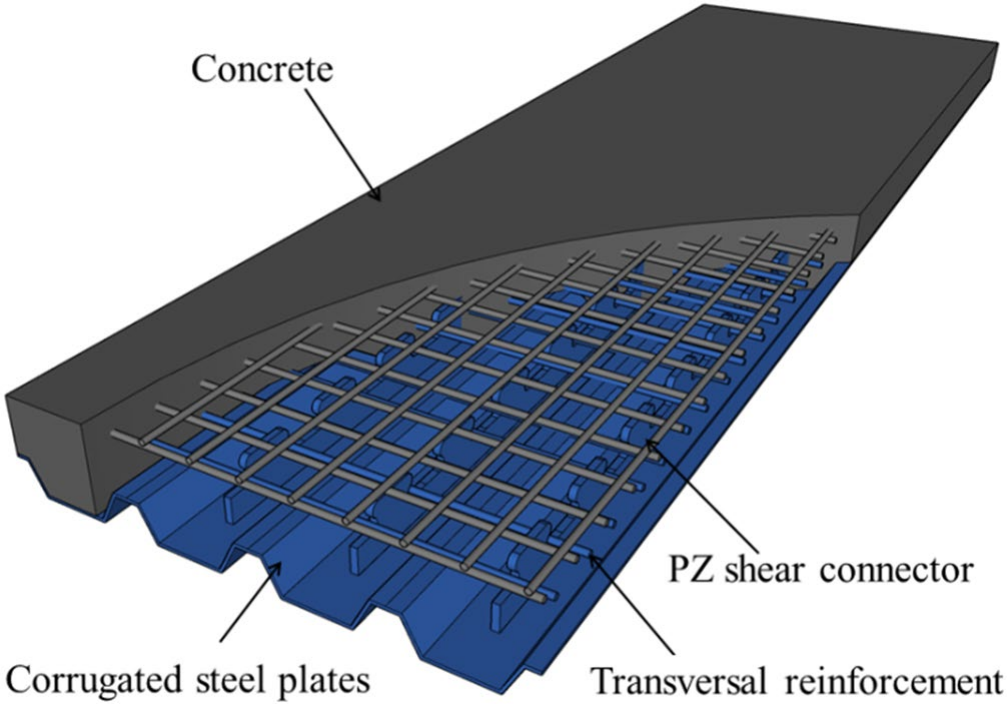
Schematic diagram of corrugated steel–concrete composite bridge decks.

Recently, steel–concrete composite bridge decks have become more widely used. Figure 1 shows a new kind of steel–concrete composite bridge deck slab. It is composed of corrugated steel sheets, concrete, and shear connectors^1–3^. Corrugated steel sheeting can be prefabricated and serve as stay-in-place (SIP) steel forms when concrete is casted on-site. Compared with reinforced concrete bridge decks, it has the advantages of high bearing capacity and fast construction^4,5^.

The steel and concrete members in the composite structure work together through the shear connector. Perfobond rib connector (PBL) is the most commonly used shear connector for creating a composite action. Di studied the mechanical properties and bearing mechanism of the large PBL connectors with strong confinement^6^. The results showed a difference of 191% in the shear stiffness of connectors with different shapes. The interaction effects in a PBL within a connector group was investigated by Guan and practical formulas were proposed for the PBL group prediction^7^. In another study, Yang studied the mechanical properties of PBL shear connectors under different bearing types. By taking the contribution of end concrete into account, the authors presented a formula for bearing capacity calculation^8^. Zhang evaluated the fatigue life of the PBL shear connector group and reported that the degree of fatigue damage in each layer of the shear connector is sensitive to the upper load level^9^. However, PBL shear connectors are not suitable for this type of bridge deck, due to their structural limitations.

Puzzle (PZ) shape composite dowel is a half-open shear connector. During construction, the transversal reinforcement is easy to install and put into the designated position. Moreover, its bearing capacity is better than the PBL shear connectors, and exhibiting good anti-fatigue performance as well^10–13^. Researchers have carried out a series of static tests and theoretical studies on PZ shape composite dowel shear connectors in recent years. Shear design approaches for pry-out failure have been proposed by Classen^14,15^, while other researchers have focused on modeling the steel failure in thin UHPC elements ^16^. Lorenc conducted full-scale push-out tests to study how the steel thickness, grade, and dowel size can affect the bearing capacity ^17^. The results proved the plastic deformation of steel dowel to be the main factor causing concrete failure. Finite element (FE) method is also used to analyze the failure mechanism of shear connectors^18^. Lacki optimized the shape of composite dowel shear connector used in a composite structure^19^. A parametric study of steel–concrete composite beam with composite dowel connectors was done by Derlatka and the best solution for composite dowel beam was selected from seven variants. Additionally, using the FE method makes it possible to investigate the influence of mechanical loads on the strains and stresses in the floor beam^20^.

However, PZ shape composite dowel shear connectors are mainly used in prefabricated composite beams in some European countries^21–26^, and these research studies are also based on composite beams. The related research and practical applications of corrugated steel–concrete composite bridge decks have not well-documented. Due to the large size and thickness of the shear connectors in composite beams and the effect of stirrups, the standard calculation of bearing capacity is not applicable to this composite bridge deck.

This study mainly aimed to evaluate the applicability of PZ shape composite dowel shear connectors with small size and weak constraints in corrugated steel–concrete composite bridge decks. This paper empirically studied the shear behavior of the connectors with elliptical holes, and applied push-out tests to investigate the static performance of PZ shape composite dowel shear connectors. Accordingly, several groups of PBL shear connectors were compared. The effects of different parameters such as transversal reinforcement, elliptical holes, and bonding friction on the bearing capacity, slip, shear stiffness, ductility, and strain of shear connectors were also investigated. Considering the practical engineering applications, the bearing capacity and load–slip formulas of PZ shape composite dowel shear connectors with elliptical holes were devised, which provides a foundation for the design of shear connectors in corrugated steel–concrete composite bridge deck. This research can pave the path for the generalization and application of PZ shape composite dowel shear connectors in China.

## Experimental program

### Design of specimens

**Figure 2:**
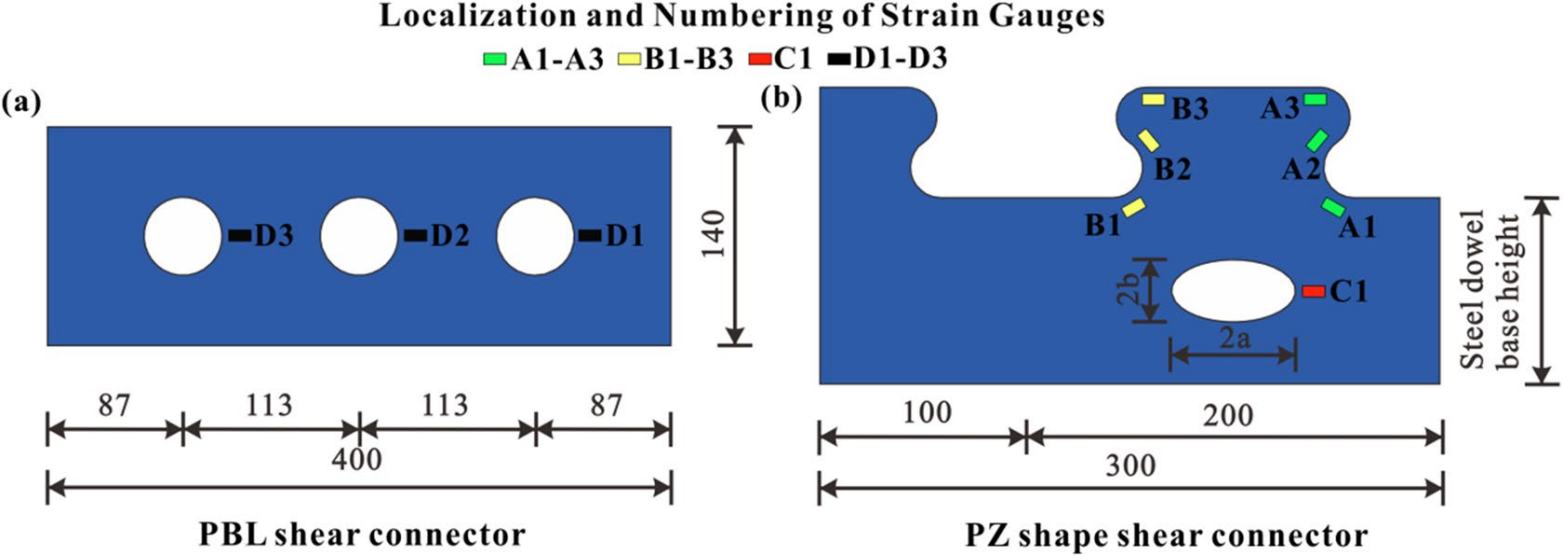
Geometry of PBL and PZ shear connectors.

**Figure 3:**
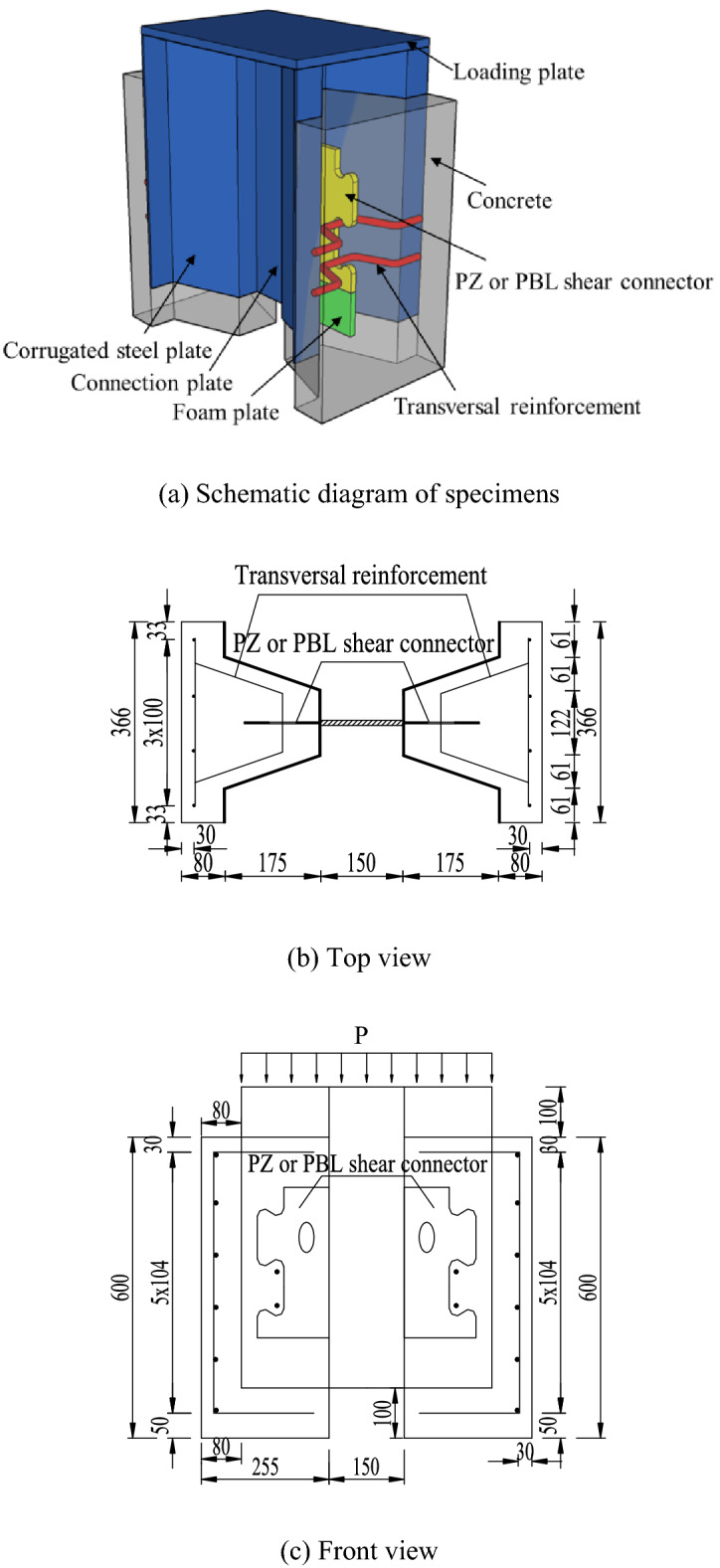
Structural dimensions of push-out specimens.

In this paper, eight specimens were designed for push-out tests. The main parameters of each specimen are shown in Tables 1 and 2. In the specimens, the thickness of corrugated steel sheeting is 6 mm, the shear connector thickness is 14 mm, and the materials for both are Q345 qE. The concrete dowel transversal reinforcement is a HRB400 bar with a diameter of 16 mm. The strength of the concrete is C50 grade. A single layer of reinforced mesh is arranged 30 mm away from the upper surface of the concrete. The reinforcement is HRB400 grade. The diameter of the reinforcement is 12 mm. The spacing between the reinforcement is 100 mm. In Table 2, a and b refer to the major and minor semi-axes of the ellipse, respectively. Geometry of the PBL and PZ shape composite dowel shear connectors are shown in Fig. 2. Structural dimensions of the push-out specimens are shown in Fig. 3.

**Table 1:** Parameters of PBL shear connector specimens.

Specimen	Hole diameter (mm)	Concrete strength (MPa)	Diameter of transversal reinforcement (mm)
PBL-1	50	54.3	–
PBL-2	50	53.6	16
PBL-3	50	54.1	16

**Table 2:** Parameters of PZ shape shear connector specimens.

Specimen	Concrete strength (MPa)	Diameter of transversal reinforcement (mm)	Ellipse dimensions (a and b) (mm)	Steel dowel base height (mm)
PZ-1	53.8	2d16	30/15	90
PZ-2	52.7	–	30/15	90
PZ-3	53.1	2d16	–	90
PZ-4	54.2	2d16	–	120
PZ-5	53.5	2d16	40/20	120

**Figure 4:**
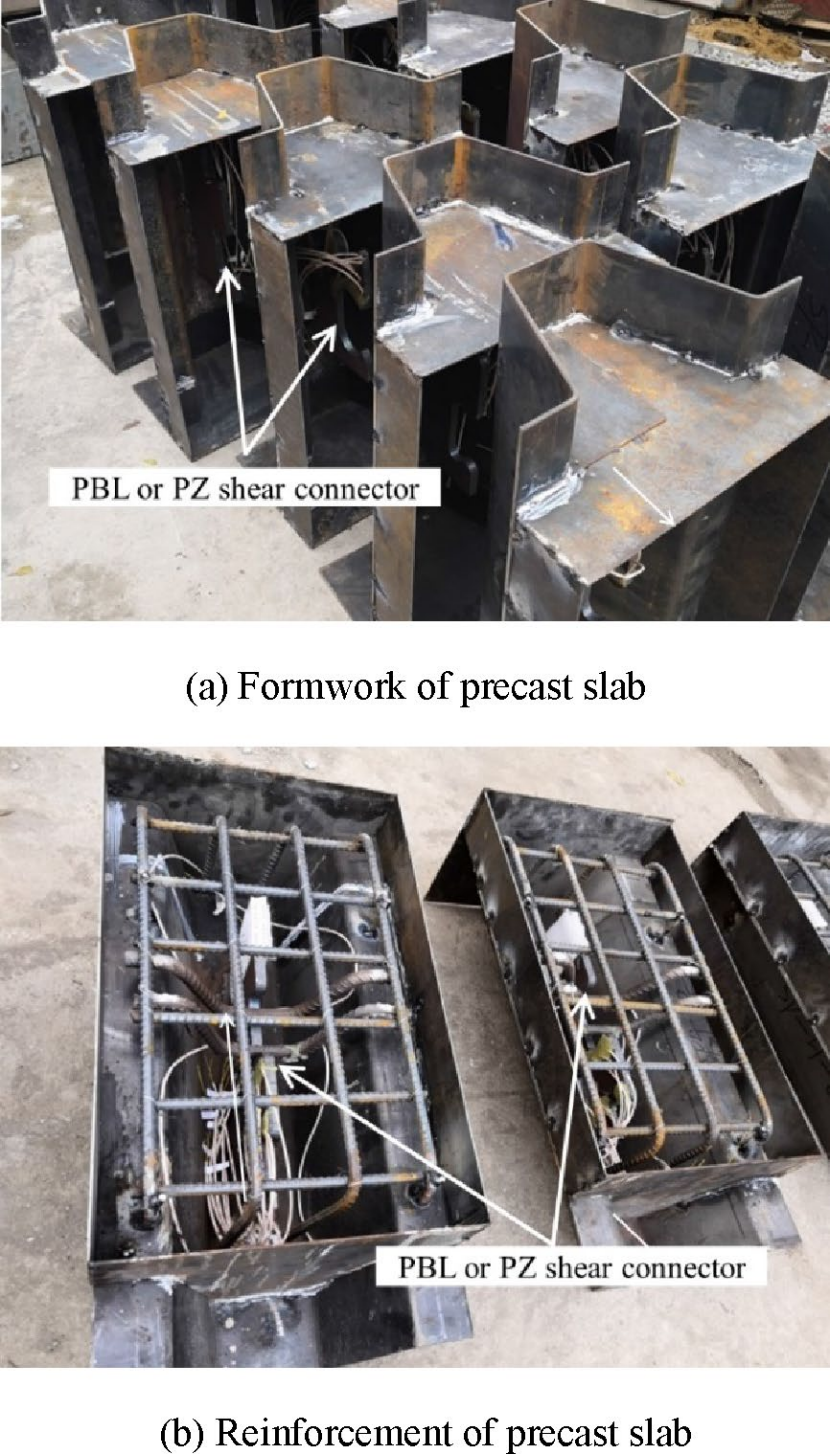
Specimens before casting concrete.

During the production of the specimen, a foam plate with a pad length of 50 mm at the bottom of the shear connector was used to eliminate the pressure effect of the concrete. With the exception of PBL-2, all other specimens were smeared with butter on the contact surface between the shear connector and concrete to eliminate the influence of a bonding force between the steel plate and concrete. Figure 4 shows the specimens before casting concrete.

### Measurement content and point arrangement

The measured parameters included the strain of the surface concrete, shear connector, and transversal reinforcement; the relative slip of the concrete and shear connector; and the load value. Figure 2 shows strain gauge layout. In this figure, A1-3, B1-3, C1 and D1-3 are the numbers of strain gauges. The relative slip was obtained by using two displacement transducers, and the strain was obtained via the static and dynamic data acquisition systems. The acquisition frequency was 1 Hz. Load values were obtained directly from the computer.

### Test program

**Figure 5:**
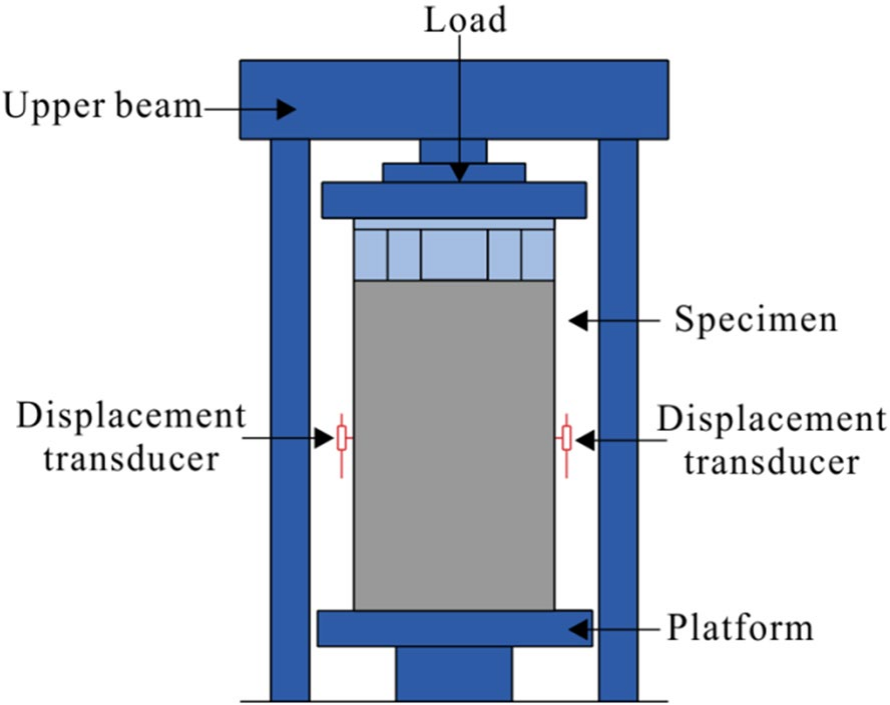
Schematic diagram of the load setup.

**Figure 6:**
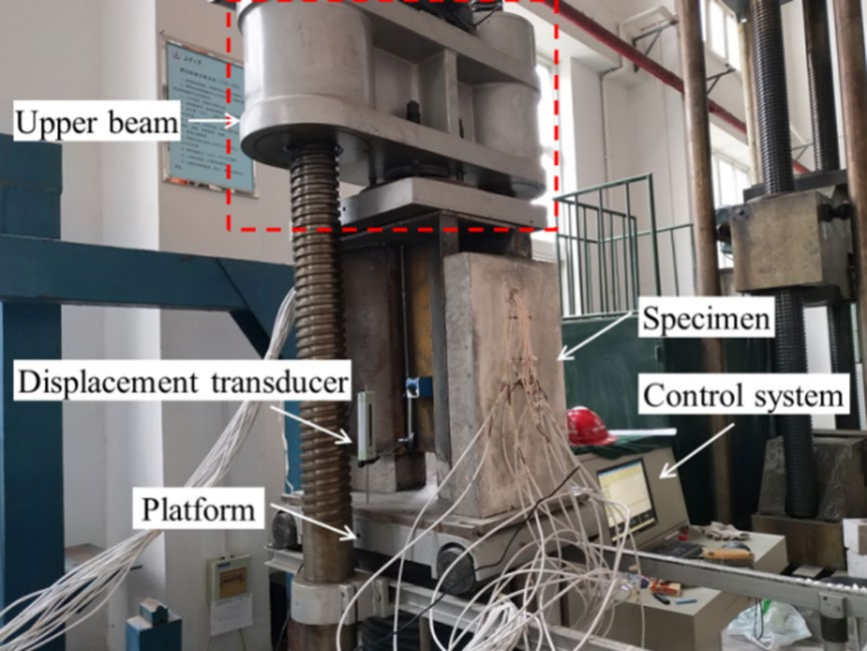
Load setup of specimens.

The loading device for this test is an electro-hydraulic servo press controlled by a 200 t microcomputer. Figure 5 schematically shows the load setup. The specimens were first placed on the loading device platform and then were subjected to the load by the upper beam. Figure 6 shows the loading setup. The test was preloaded three times before formal loading to eliminate inelastic deformation and to verify normal operation of the acquisition system. Formal loading was divided into two stages: load control and displacement control. The loading rate is 2 kN/s under load control. The loading rate is 0.2 mm/s under displacement control.

The mechanical properties of the main materials were tested before the push-out test. Tables 1 and 2 show the strength of the concrete, and the test results of the transversal reinforcement and steel plate are presented in Table 3.

**Table 3:** Test results of material properties.

Material	Yield strength (MPa)	Ultimate strength (MPa)
Steel plate (Q345Qe)	422.6	524.1
Transversal reinforcement (HRB400)	446.0	604.8

## Results and discussion

### Failure mode

**Figure 7:**
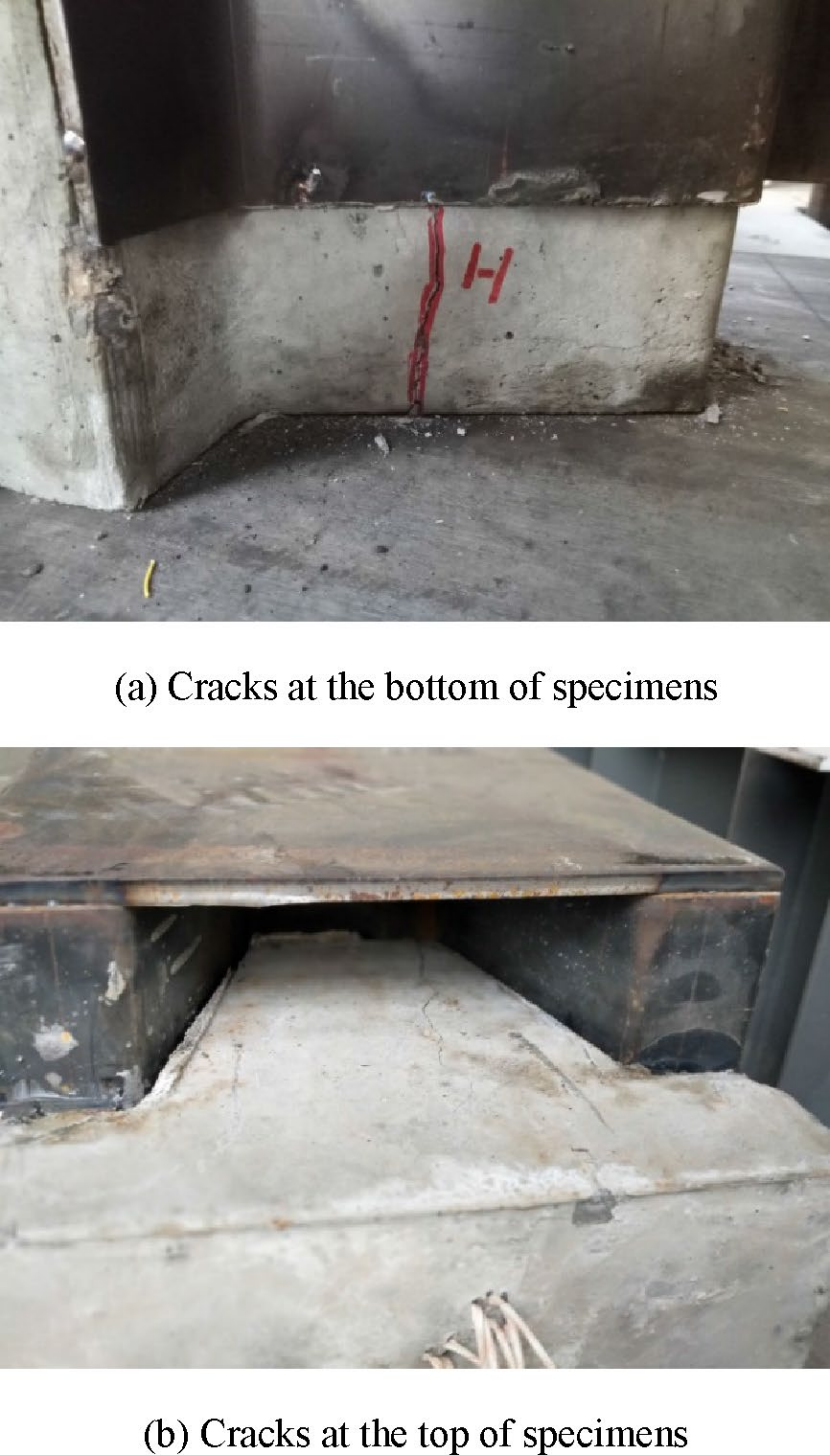
Concrete cracks in a specimen during testing.

At initial loading, there was no obvious slip. With the increase of load, the displacement transducers showed slip of the steel plate and concrete, but the specimens showed no obvious visible changes. After loading to the limit load of 80%, the slip visibly increased. Firstly, several vertical micro-cracks were observed at the bottom of the specimen, which extended from the inside of the specimen. Subsequently, the crack continued to expand and gradually widened. At the same time, cracks appeared on the sides and top of the concrete. Finally, the top and bottom cracks were connected vertically. The failures exhibited are all concrete shear failure. The concrete at the bottom of the specimens had different degrees of spalling. The sound of concrete cracking was noted during testing. For the specimens without transversal reinforcement, the load changed clearly in the descending section. Figure 7 shows an example of the concrete cracks in a specimen during testing.

**Figure 8:**
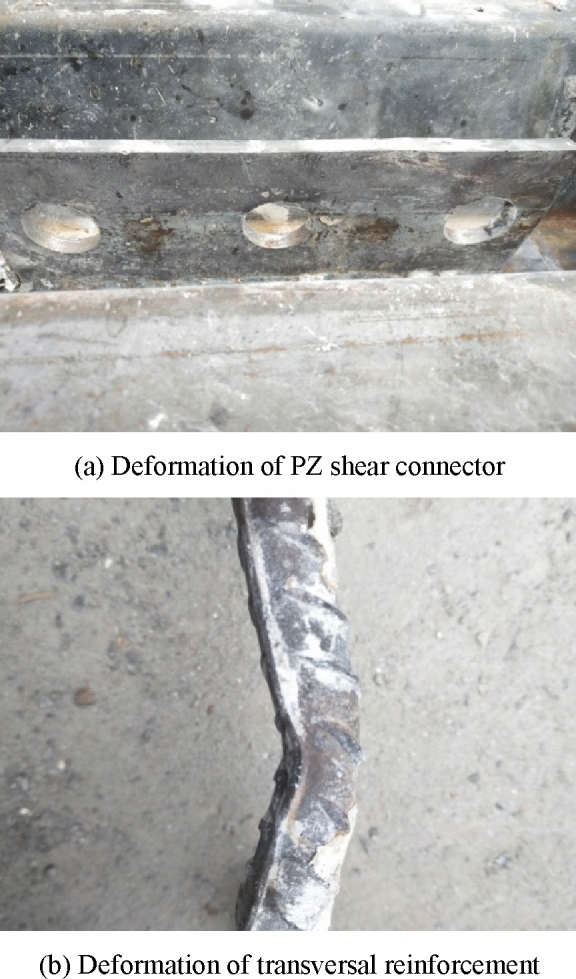
A specimen with PBL shear connector after breaking.

**Figure 9:**
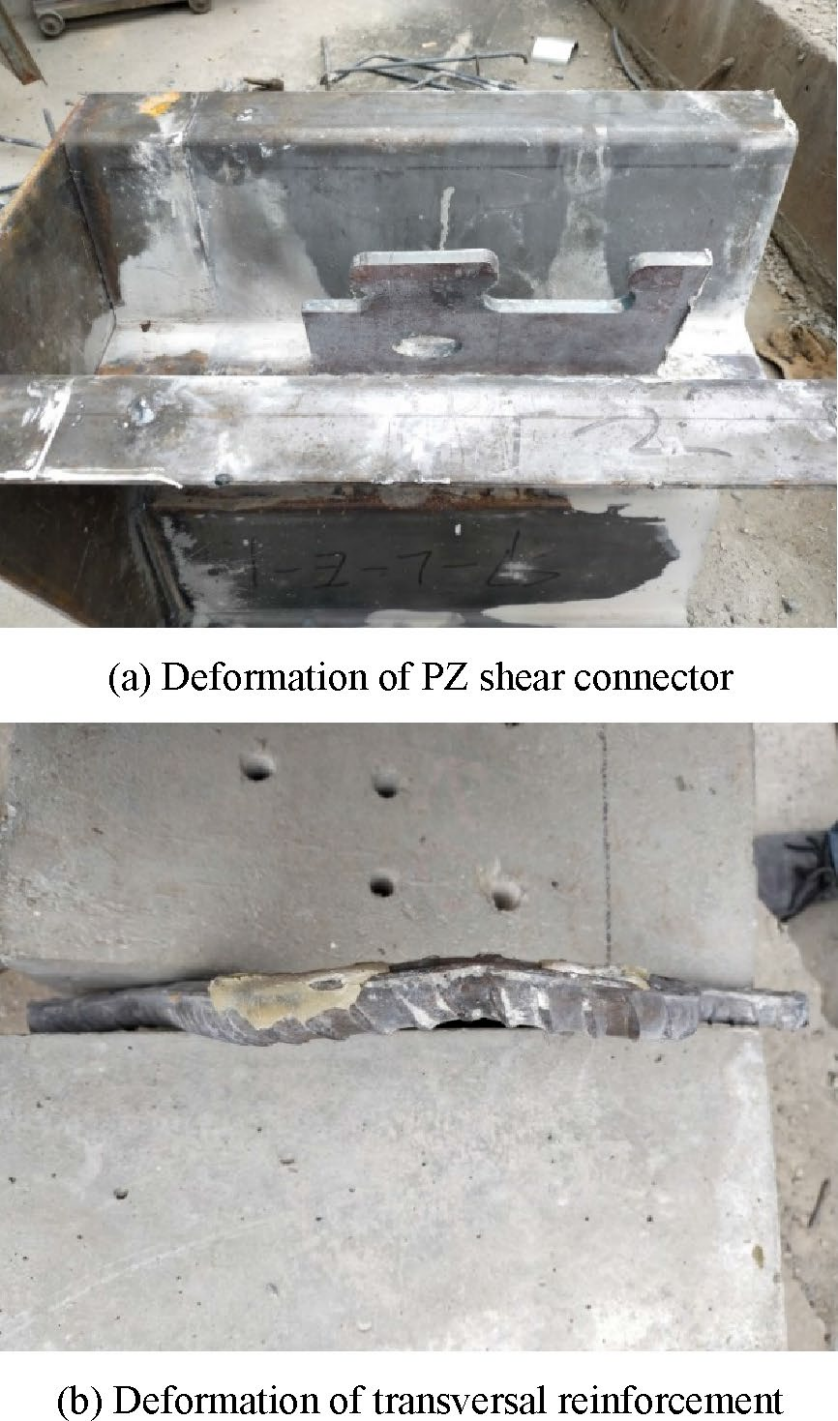
A specimen with PZ shear connector after breaking.

Figures 8 and 9 illustrate some examples of deformed specimens with PBL and PZ shape shear connectors after breaking, respectively. It can be seen from the figures that no clear deformation or failure was observed at the round holes of the PBL shear connector. Similarly, the steel dowels and elliptical holes of the PZ shape composite dowel shear connector showed no obvious deformation. However, the transversal reinforcement of the two shear connectors was clearly deformed, but it was not cut. This is because the specimen has large slip, so the transversal reinforcement was squeezed to the edge of the round hole and steel dowel, and the concrete and dowel bear the shear force together. By comparing the two different shear connectors, since in the PBL connectors, the transversal reinforcement was closer to the round holes, the deformation of the transversal reinforcement in the PBL shear connector was more obvious than that of the PZ shape one. Double plane shearing of the concrete dowel was the dominant failure mode in both connector types.

### Load–slip curves and static mechanical property indexes

**Figure 10:**
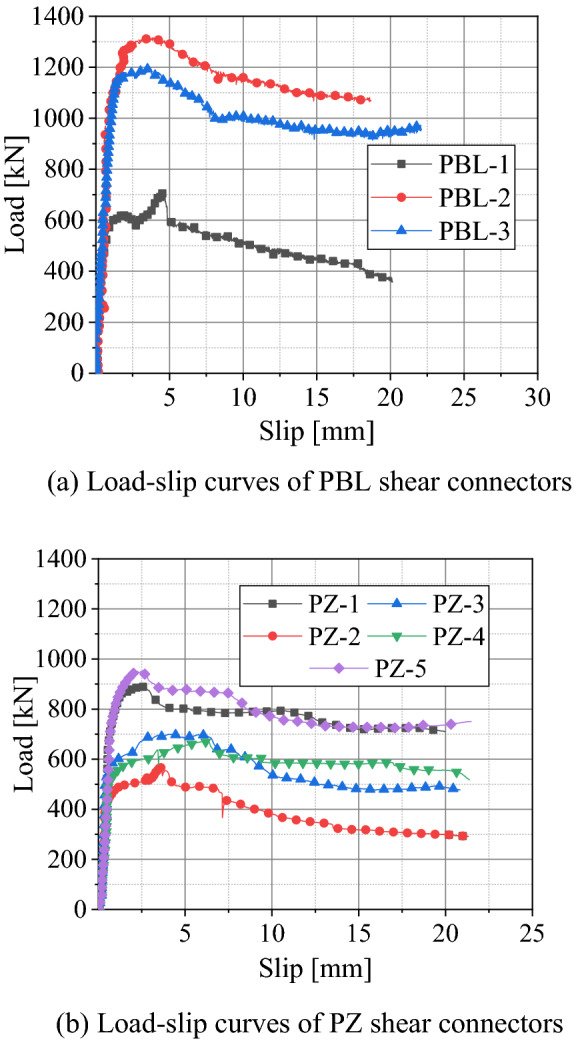
Load–slip curves of specimens.

The load–slip curves and static mechanical property indexes of the test specimens are shown in Fig. 10 and Table 4, respectively. The main static mechanical property indexes of shear connectors are as follows: bearing capacity limit value *P*_*u*_, characteristic bearing capacity *P*_*Rk*_, bearing capacity design value *P*_*Rd*_, limiting slip *δ*_*u*_, design slip *δ*_*d*_, shear stiffness *K*_*s*_, and ductility factor *D*_*c*_. *P*_*u*_ is the maximum bearing capacity of the shear connector. *P*_*Rk*_ is 0.9 *P*_*u*_, and *P*_*Rd*_ is *P*_*Rk*_ divided by the factor of *γ*_*v*_, where *γ*_*v*_ = 1.25. *δ*_*u*_ is the maximum value of the slip amount corresponding to *P*_*Rk*_, *δ*_*d*_ is the slip amount corresponding to *P*_*Rd*_. *K*_*s*_ is the slope of the line between the origin and *P*_*Rd*_ on the load–slip curves. *D*_*c*_ is the ratio of *δ*_*u*_ to *δ*_*d*_^27^. The values in Table 4 have been converted to the corresponding test values of a single hole and a single steel dowel.

**Table 4:** Static mechanical property indexes of tested shear connectors.

Specimen	*P*_*u*_ (kN)	$\delta$_*u*_ (kN)	*P*_*Rk*_ (kN)	*P*_*Rd*_ (kN)	$\delta$_*d*_ (kN)	*K*_*s*_ (kN mm^−1^)	*D*_*c*_
PBL-1	116.4	4.817	104.8	83.8	0.667	125.6	7.2
PBL-2	219.2	8.233	197.3	157.8	0.725	217.6	11.4
PBL-3	200.2	7.393	180.2	144.1	0.761	189.4	9.7
PZ-1	428.3	10.786	385.5	308.4	0.546	564.8	19.8
PZ-2	283.8	4.359	255.4	200.3	0.379	528.5	11.5
PZ-3	333.1	7.873	299.8	239.8	0.403	595.0	19.5
PZ-4	318.8	9.451	286.9	229.5	0.393	584.0	24.0
PZ-5	459.7	7.938	413.7	331.0	0.643	514.8	12.3

### Ultimate bearing capacity

The ultimate bearing capacity of PBL-3 increased by 72.0% compared with that of PBL-1, and the ultimate bearing capacity of PZ-1 increased by 60.3% compared with that of PZ-2. The only difference between PBL-3 and PBL-1 is that PBL-3 has transversal reinforcement. Likewise, the only difference between PZ-1 and PZ-2 is that PZ-1 has transversal reinforcement. It can be seen that transversal reinforcement can significantly improve the bearing capacity of the specimen.

The ultimate bearing capacity of PBL-2 was 19 kN higher, or about 9% higher, than that of PBL-3. Therefore, the presence of a bonding force and friction force between the steel plate and concrete creates a measurable difference in shear properties. However, due to the complex working mechanisms and unpredictable performance of the bonds, it is usually considered as a safety measure and is not included in the calculation of the ultimate bearing capacity.

In order to compare the ultimate bearing capacity of PZ and PBL shear connectors, the shear connectors were converted into lengths of 400 mm. The ultimate bearing capacity of PZ-3 is 65.6 kN higher, or 11% higher, than that of PBL-3. The comparison between PZ-1 and PZ-3 shows that the ultimate bearing capacity of elliptical hole is about 95 kN. Thus, it can be shown that the ultimate bearing capacity of PZ-2 is about 188.8 kN if no elliptical hole is opened. When an elliptical hole is not opened, and the length of shear connector is 400 mm, the ultimate bearing capacity of PZ-2 increases by 28.4 kN, or 8.1%, when compared with that of PBL-1. Therefore, the ultimate bearing capacity of the PZ shear connector is higher than that of PBL shear connector, with or without transversal reinforcement penetration.

The ultimate bearing capacity of PZ-3 increased by 14.4 kN, or 0.04%, when compared with PZ-4. This shows that the ultimate bearing capacity decreases slightly with the increase of steel dowel height. The ultimate bearing capacity of PZ-1 increased by 95.2 kN, or 28.6%, compared with that of PZ-3, and the ultimate bearing capacity of PZ-5 increased by 126.5 kN, or 38.0%, compared with that of PZ-3. It can be seen that the bearing capacity can be improved by opening elliptic holes in the composite dowel shear connector.

### Shear stiffness and ductility

It can be seen from Table 4 that the shear stiffness of the PZ shear connectors is greater than that of PBL shear connectors. By comparing PBL-3 with PBL-1, and PZ-1 with PZ-2, it is shown that transversal reinforcement can effectively improve the shear stiffness of shear connectors. The shear stiffness of PZ-5 is smaller than that of PZ-1. The shear stiffness of PZ-1 and PZ-5 are both smaller than that of PZ-3 and PZ-4. The results show that the shear stiffness of shear connectors will be reduced by opening elliptical holes, while the reduction degree with large holes is greater than that with small holes. Although opening elliptical holes can increase the bearing capacity of PZ shape composite dowel shear connectors, it can also reduce the shear stiffness. Therefore, the balance between the two should be fully considered in the design of shear connectors, and the size of elliptical holes cannot be increased without limit.

Ductility factor is also an important index to reflect the shear connector performance. Shear connectors should have good ductility when they are broken. In EN 1994-1-1, a minimum deformation capacity of 6 mm is demanded for ductile shear connectors^28^. The *δ*_u_ values for PBL-1 and PZ-2 specimens were below 6 mm, while it was above 6 mm for the other specimens. It is worth noting that the transversal reinforcement was the difference between specimens PBL-1 and PZ-2 and other specimens where these two samples had no transversal reinforcement. Therefore, it can be concluded that transversal reinforcement enhanced the ductility of shear connectors. Additionally, the ductility factor of PZ shear connectors was shown to be higher than that of PBL shear connectors, resulting in a higher ductility of PZ shear connectors.

### Load–strain curves

**Figure 11:**
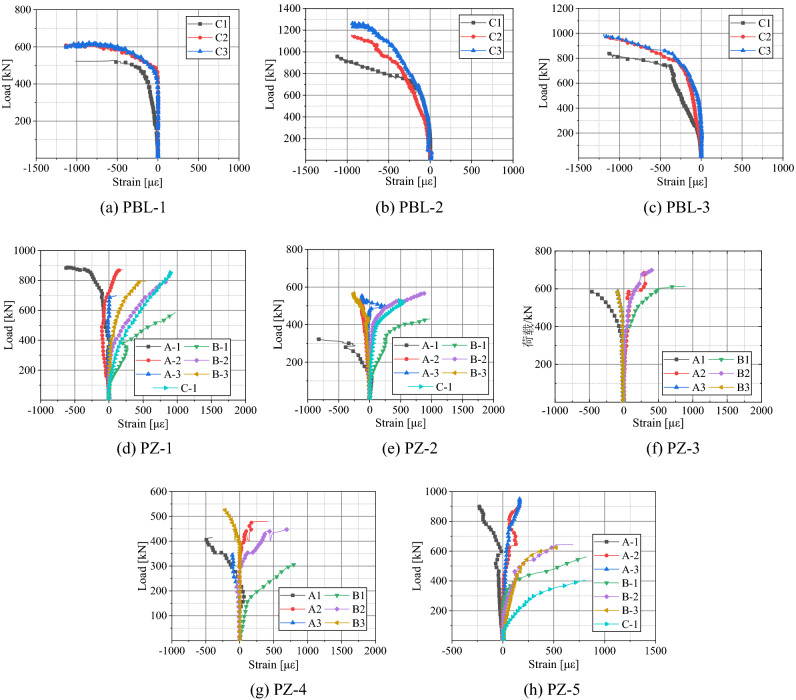
Load–strain curves of steel dowels.

**Figure 12:**
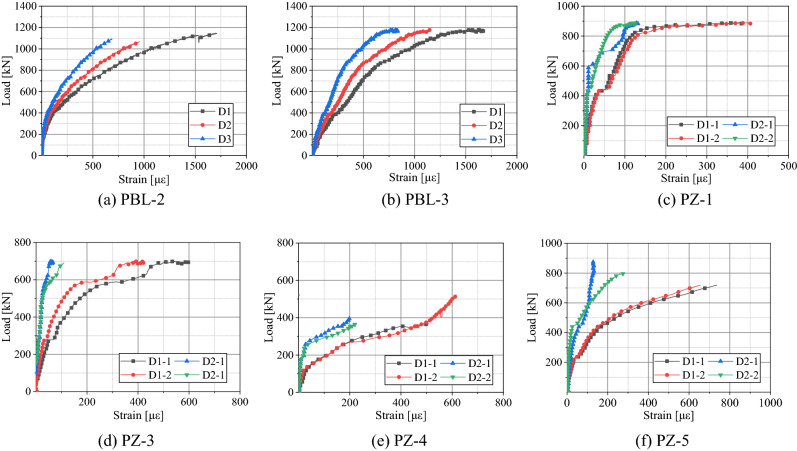
Load–strain curves of transversal reinforcement.

The concrete surface showed no obvious visible strain. This is due to the gap between the top of the shear connector and the concrete surface, between which there are structural bars. The failure mode is not concrete pry-out. Therefore, the load–strain curves of the concrete are not listed in this paper. Figure 11 shows the load–strain curves of the steel dowels and the transversal reinforcement load–strain curves are depicted in Fig. 12.

As can be seen in Fig. 11, the load–strain curves of the two types of shear connectors have a linear relationship with the initial loading. For the PBL-1 specimen, when the load value was 490 kN, or about 0.7 *P*_u_, the microstrain values at point C1 and C3 were about 303 and 110, respectively. The value at point C1 was about 2.8 times that of point C3. Regarding the PBL-2 specimen, the microstrain value at point C1 was about 1020, which was about three times that of point C3, with a value of 340, when the load value was 920 kN, or about 0.7 *P*_u_. Likewise, at a load of 840 kN, or about 0.7 *P*_u_, for the PBL-3 specimen, the microstrain value at point C1 (approximately 1140) was about three times that of point C3 (approximately 380). It can be concluded that for all the three PBL shear connectors, the most changes appeared in C1, which was the closest to the loading point, while the C3, the farthest area from the loading point, experienced the least significant changes. In all measuring points of PZ shear connectors, the A and B areas were under compressive and tensile stresses, respectively. The strain change is the most obvious at A1 and B1 (lower part of the steel dowel). The strain increases rapidly with the increase of load. A1 and B1 are regions of stress concentration. The strain changes at A3 and B3 are relatively insignificant.

As shown in Fig. 12, the transversal reinforcement strain was negligible during the initial loading. D1 was the closest to the loading point among all strain measuring points, followed by D2 and D3. It can be claimed that as the load increases, the concrete pins are gradually crushed, and the transversal reinforcement begins to take the load, resulting in a sharp increase in the strain. The results showed a gradual bend of the transversal reinforcement. For the PBL-2 specimen at a load of 920 kN, the microstrains at points D1, D2, and D3 were 880,655 and 435, respectively. Moreover, when the load of PBL-3 was 840 kN, the microstrain values of 660,490, and 320 were measured at points D1, D2, and D3, respectively. The load–strain curve of D1 had a more significant change than other measured points. This showed that the concrete tenon at D1 was crushed first, and the transversal reinforcement was stressed earlier. Comparing the PBL-2 and PBL-3 specimens, the PBL-3 showed higher values of initial strain of the transversal reinforcement. The adhesion and friction between the concrete and the steel plate at the initial loading stage play an important role in resisting the load. As the PBL-2 was not coated with butter, lower values of strain were observed. For specimens with PZ shape composite dowel shear connectors, when the load value was 0.7 *P*_u_, the microstrain value of point D1 was about four times that of point D2. The transversal reinforcement close to the loading point was mainly used to bear the load while providing a pinning force was the primary use of the transversal reinforcement far away from the loading point.

## Design recommendations

### Shear bearing capacity

Studies have shown three types of possible failure modes in PZ shape composite dowel shear connectors under load^29–31^. Different failure modes have different calculation formulas for bearing capacity.

For small openings between the dowels and large steel plate thicknesses, the dominating failure mode is the double plane shearing of the concrete dowel. Therefore, the main parameters for the load-bearing capacity are the shear area of the concrete dowel and the shear strength of the concrete. With large openings, the two shear planes merge together, which is taken into account by a geometry-dependent reduction factor *η*_*D*_. The bearing capacity is expressed by:

1 $$ p_{sh,k} = \eta_{D} \cdot e_{x}^{2} \cdot \sqrt {f_{ck} } \cdot (1 + \rho_{D} ) $$

where $\eta_{D}$ = 2 − *e*_*x*_/400, $\rho_{{\text{D}}}$ = $(E_{s} \cdot A_{b} )/(E_{cm} \cdot A_{D} )$, *A*_*b*_ is the cross-sectional area of transversal reinforcement, *A*_*D*_ is the shear area of concrete dowel (*A*_*D* _= 0.13*e*_*x*_^2^), *E*_*s*_ is the elastic modulus of transversal reinforcement, *E*_*cm*_ is the elastic modulus of concrete, and *f*_*ck*_ is compressive strength of concrete.

According to the experimental results, the failure mode of PZ shape composite dowel shear connectors in this test is concrete shear failure. Since this test design is with a single dowel, the factor *η*_*D*_ is not considered in the formula. According to Formula (2), the characteristic bearing capacity is *P*_*sh,k*_ = 329.7 kN. Using PZ-3 and PZ-4 as examples, according to the test results, the characteristic bearing capacities of PZ-3 and PZ-4 are 299.8 kN and 286.9 kN, respectively. The values calculated by the theoretical formula are larger than the experimental results.

The thickness and spacing of steel dowels are large in prefabricated composite beams. The influence of stirrups in composite beams on bearing capacity is also significant. The bearing capacity depends on the structure. The theoretical formula for prefabricated composite beams does not apply to composite bridge decks. Therefore, it is necessary to establish a formula for calculating the bearing capacity of PZ shape composite dowel shear connectors with elliptic holes in corrugated steel–concrete composite bridge decks.

The bearing capacity of PZ shape composite dowel shear connectors with elliptical holes is mainly dependent on the composite dowel and concrete tenon with elliptical holes. The bearing capacity can be expressed by:

2 $$ p_{sh,k} = p_{c} + p_{e} $$

Refer to the existing formula for the bearing capacity of composite dowels (*p*_*c*_), we introduce a reduction factor $\varphi_{c}$. According to the characteristic bearing capacity of PZ-3 and PZ-4, the value of $\varphi_{c}$ is 0.889. Then the bearing capacity of the composite dowel part is:

3 $$ p_{c} = 0.889e_{x}^{2} \cdot \sqrt {f_{ck} } (1 + \rho_{D} ) $$

According to the literature^32^, the bearing capacity of the concrete tenon (*p*_*e*_) can be expressed by:

4 $$ p_{e} = 2\varphi_{e} A_{e} \sqrt {f_{ck} } $$

where *A*_*e*_ is the area of the elliptical opening. *A*_*e*_ = $\pi ab$, where a and b are the long and short semi-axes of the elliptical hole. $\varphi_{e}$ is the influence coefficient. According to the PZ-1 and PZ-3 tests, the characteristic bearing capacity of small elliptical holes can be calculated as 86 kN. According to the PZ-4 and PZ-5 tests, the characteristic bearing capacity of large elliptical holes can be calculated as 127 kN. By substituting into Formula (6), the value of $\varphi_{e}$ is 4.738. Therefore, bearing capacity of the concrete tenon is

5 $$ p_{e} = 9.476A_{e} \sqrt {f_{ck} } $$

_In summary, the bearing capacity formula of PZ shape composite dowel shear connectors with elliptical holes in corrugated steel–concrete composite bridge decks is:_

6 $$ p_{sh,k} = 0.889e_{x}^{2} \cdot \sqrt {f_{ck} } (1 + \rho_{D} ) + 9.476\pi ab\sqrt {f_{ck} } $$

**Figure 13:**
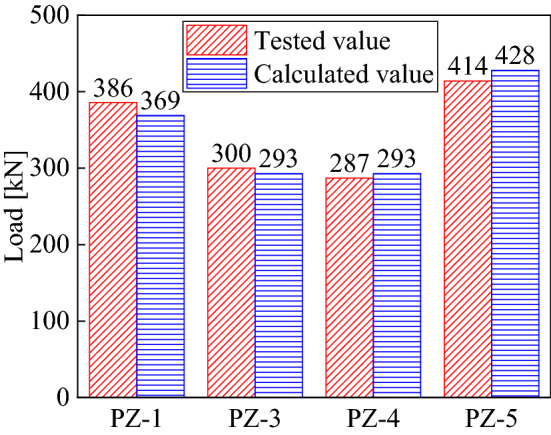
Comparison results of calculated and tested values.

According to the proposed theoretical calculation formula, the shear capacity of four tested specimens with perforated transversal reinforcement was calculated. The comparison between the calculated results and the test results is shown in Fig. 13. The errors between calculation and tested values were less than 5%, suggesting good agreement between theoretical and experimental values.

### Load–slip curve

**Figure 14:**
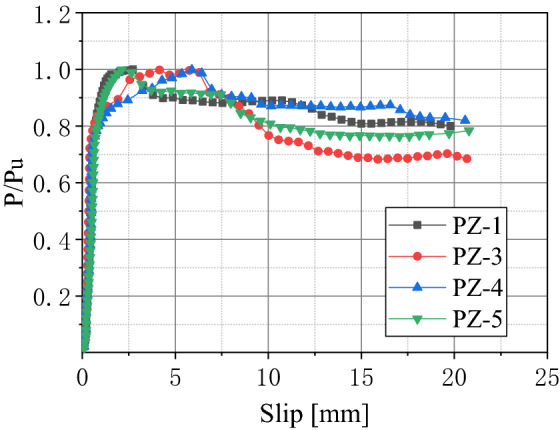
Dimensionless load–slip curve.

The load–slip curves of the PZ-1, PZ-3, PZ-4, and PZ-5 specimens were treated as being dimensionless, which are shown in Fig. 14.

It can be seen from Fig. 14 that there are two distinct areas in the dimensionless load–slip curves: rising and falling sections. The logarithmic function can be used to describe both the rising and the falling parts. The fitting curve can be expressed by:

7 $$ P/P_{{\text{u}}} = a\ln (S) + b $$

The linear regression method was used to fit the dimensionless load–slip curves of PZ-1, PZ-3, PZ-4, and PZ-5 specimens, and to understand the load–slip relationship of the shear connector in the two areas. Given this, the a and b variables were calculated as follows: in the ascending section a = 0.339 and b = 0.810, and for the descending section a = − 0.078 and b = 1.041. Therefore, the calculation formula of the load–slip curve of PZ shape composite dowel shear connector is described as:

8 $$ P/P_{u} = \left\{ {\begin{array}{*{20}l} {0.339\ln (S) + 0.810} \hfill & {0 < S \le S_{u} } \hfill \\ { - 0.078\ln (S) + 1.041} \hfill & {S > S_{u} } \hfill \\ \end{array} } \right. $$

where *P*_u_ is the ultimate bearing capacity and *S*_u_ is the corresponding slip amount under the ultimate bearing capacity.

**Figure 15:**
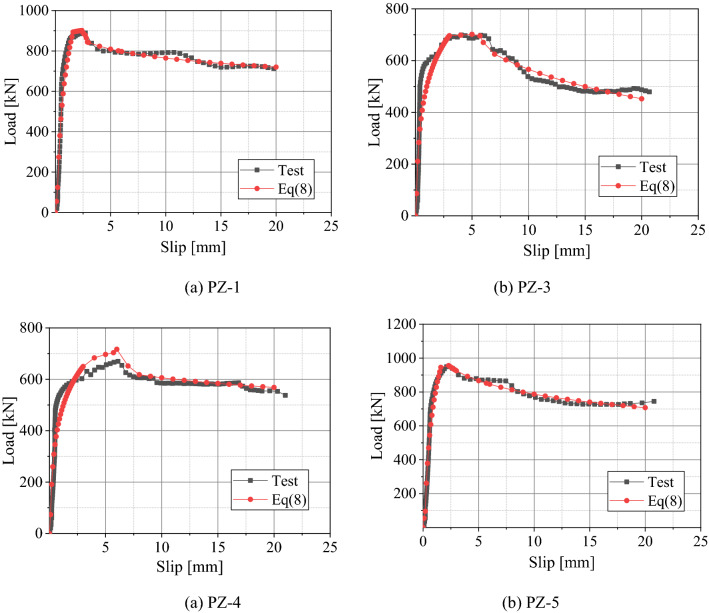
Comparison of load–slip curves between the test results and the fitting formula.

Figure 15 compares the fitted curve and the curve obtained from the test results. It can be seen from Fig. 15 that, the curve fitted according to the proposed formula is in good agreement with the one obtained from the test results. However, the calculated stiffness of the plastic region falls slightly below the measured one. Therefore, the load–slip curve proposed in this study accurately reflects the overall characteristics of the load–slip curve of the PZ shape composite dowel shear connectors.

Different mechanical features of a shear connector, such as the bearing capacity, shear stiffness and deformation capacity can be obtained from the load–slip curve, and is the most important mechanical feature of the shear connector. Transversal reinforcement and concrete tenons determine the bearing capacity of a PZ shape composite dowel shear connector. The bearing capacity provided by concrete tenon is affected by various factors such as dowel size, concrete strength and constraint state. In contrast, for the transversal reinforcement, the bearing capacity varies significantly under different constraints. PZ shape composite dowel shear connectors are known to have a complex bearing mechanism. The calculated values for the bearing capacity are quite different, with the measured ones in the push-out test, due to the different factors considered by researchers. The theoretical formulas in this work are applicable to the PZ shape composite dowel shear connectors with small size and weak constraints in the corrugated steel–concrete composite bridge deck. However, as the experiments were performed only once, there was no test redundancy. Further studies with more tests are required to achieve reliable results.

## Conclusion

The push-out tests of PBL and PZ shape composite dowel shear connectors with elliptical holes in corrugated steel–concrete composite bridge decks were conducted, and their mechanical behavior was also studied. Based on the test results in this paper, several conclusions can be drawn and are summarized as follows.

1. The two exhibited failure forms were shear failure of concrete and ductile failure of shear connectors. In the case of failure, there was no significant strain change in the steel dowel and elliptical hole of the PZ shape shear connector or the round hole of the PBL shear connector. But there was obvious deformation in the transversal reinforcement in both designs.
2. Both types of shear connectors had the same load–slip tendency. Transversal reinforcement is an important factor affecting bearing capacity and shear stiffness. The ultimate bearing capacity of shear connectors can be significantly increased by opening elliptical holes. But this will also reduce the shear stiffness. The shear connector height has little effect on the bearing capacity.
3. In corrugated steel–concrete composite bridge decks, PZ shape composite dowel shear connectors are superior to PBL shear connectors in bearing capacity, shear stiffness, and ductility. Due to the structural characteristics of the PZ shape shear connector, it is easy to penetrate the transversal reinforcement.
4. The comparative analysis of the existing theoretical calculation results and experimental results shows that the theoretical calculation results are slightly higher. The calculation formula of PZ shape composite dowel shear connectors in composite beams in European technical standards is not applicable to composite bridge panels. In this paper, based on the existing research and considering engineering practice, a formula for calculating the bearing capacity of PZ shape composite dowel shear connectors with elliptic holes in corrugated steel–concrete composite bridge decks was proposed. Based on the characteristics of load–slip curves obtained from experiments, two formulas were proposed for the load–slip curves and were used to fit a curve to the experimental data. The results calculated by the formulas agreed well with the experimental results. These two formulas may be used to guide the design of shear connections in corrugated steel–concrete composite bridge decks.

In the present paper, an experimental study was done on the behavior of PZ shape composite dowel shear connectors, yet the theoretical investigations were limited. A further detailed analysis of the FEM simulations will be presented in a separate study.
